# Activate dormant C–H bonds with tons of enthusiasm: an interview with Jin-Quan Yu

**DOI:** 10.1093/nsr/nwab229

**Published:** 2021-12-28

**Authors:** Weijie Zhao (赵维杰)

**Affiliations:** NSR news editor based, Beijing

## Abstract

Synthetic chemistry is the art of molecules. By breaking and forming chemical bonds, chemists can transform one molecule into another, and provide a variety of chemicals that we use in our daily lives. Among all chemical bonds, the carbon-hydrogen (C–H) bond is one of the most common, and is present in almost every organic compound. Breaking C–H bonds and connecting the carbon atoms with other atoms or groups is an essential step for the synthesis of a large variety of chemicals—from bulk chemicals to functional materials and drug candidates. However, the C–H bond is extremely stable and difficult to break, and C–H activation and functionalization has been a challenging fundamental problem for decades.

Professor Jin-Quan Yu (余金权) from the Scripps Research Institute is one of the most active scientists in this field. His group developed a number of C–H activation catalysts—often consisting of metal centers and elaborately designed ligands—that drastically shorten the synthesis steps of diverse functional molecules and provide high reactivity and selectivity. Here, Professor Yu discussed with *NSR* the interesting field of C–H activation, as well as sharing reflections on his own research.

## C–H ACTIVATION: ADVANCES AND CHALLENGES

***NSR:*** In the field of C–H activation, what have been the major landmarks over the past 20 years, including your contributions?

***Yu:*** To evaluate advances in this field, it is essential to divide C–H activation into two subfields: first, *initial functionalization of alkane*, mainly methane oxidation for fuel transportation or alkane functionalization for bulk chemical production; and second, *further functionalization of synthetically relevant substrates*, for making complex molecules with new functions. The measures of success in these two subfields are different, and the approaches for solving these two problems are fundamentally different. Confusing these two distinct aspects has been one of the major obstacles that might have impeded the advance of C–H activation chemistry, especially for the subfield of further functionalization. A logical analysis and in-depth deliberation of what are the challenges for developing synthetically useful reactions was the first important step we took in 2002.

For the oxidation of methane or alkane, the challenge is less about reactivity or selectivity, because excess substrate under high pressure or temperature could be used to drive the reaction. Typically, only one or two types of C–H bonds are involved, so the main selectivity issue is to avoid overoxidation. Only one good reaction is needed in this case. Instead, the most crucial challenge is efficiency and cost as they produce relatively cheap bulk chemicals that are used as fuel or raw materials for chemical synthesis. Unfortunately, progress in addressing this challenge has been very slow in the past 30 years. It is reasonable to suggest that this problem will be best addressed by heterogeneous or enzymatic catalysis. The catalysts have to be as robust as, and as cheap as, zeolites.

**Figure 1:**
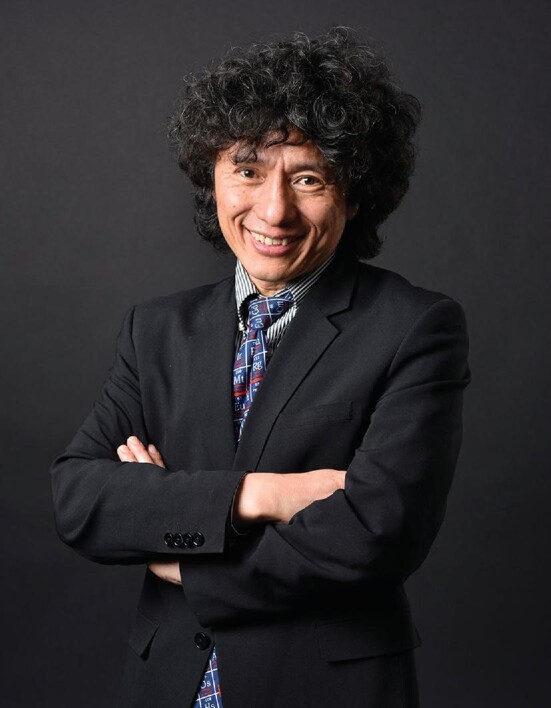
Jin-Quan Yu is one of the most active scientists in the field of C–H activation (*courtesy of Prof. Jin-Quan Yu*).

For further C–H functionalization of native substrates such as aliphatic acids, ketones, amines and alcohols, the reactivity of one equivalent of substrate under mild conditions is a tremendous challenge. Selectivity is also a great challenge, as almost all C–H bonds in these types of substrates are different due to the presence of one or two functional groups, therefore the activation of each of these C–H bonds will lead to different synthetic disconnections. In addition, one transformation will be far from being sufficient, since each synthetic step may require formation of a different bond in order to achieve the ideal synthesis. To systematically advance this subfield, we identified four core challenges to focus upon, which I call the 4Ys:

1. Reactivit**Y**: diverse native substrates and diverse carbon-carbon and carbon-heteroatom bond formations;
2. Enantioselectivit**Y**: controlling the chirality of the C–H metalation step by desymmetrizing tetrahedral carbon centers;
3. Site-selectivit**Y**: selective activation of multiple remote C–H bonds in the absence of conventional electronic or steric effects;
4. Sustainabilit**Y**: achieving catalytic turnovers using scalable green oxidants such as molecular oxygen or aqueous hydrogen peroxides as the terminal oxidants.

Major advances in addressing these four challenges have been made in the past 10 years, largely due to the invention of several generations of new ligand-based bifunctional catalysts in our laboratory.

***NSR:*** What are the catalysts designed by your lab that made progress in addressing the challenges of the 4Ys?

***Yu:*** Our first breakthrough was the discovery of a bifunctional Pd(II) catalyst based on mono-protected amino acids (MPAA) in 2008. When we observed that this MPAA ligand can drastically accelerate C–H activation as well as promote a diverse range of subsequent formation of carbon-carbon and carbon-heteroatom bonds, we knew we had hit upon a game changer. It took us a few years to figure out that this ligand is actually bifunctional. Typically, ligand tunes the property of a metal center by coordination in catalysis. In contrast, the MPAA ligand has an additional role that is vital: the mono-protected amino group directly participates in the transition state and cleaves the C–H bonds. Based on this fundamental understanding, we have developed five new generations of ligands: MPAQ, MPAO, MPAAm, MPAThio, and most recently the pyridine-pyridone (PyriPyri) ligand. These chiral ligands have also enabled us to realize enantioselective C–H activation via asymmetric metalation for the first time, after a long pursuit over the past five decades.

***NSR:*** Have your reactions been used in industry?

***Yu:*** Many. In fact, my chair professorship was endowed by one of the leading pharma companies Bristol-Myers Squibb, my collaborator. For example, one of our C–H lactonization reactions has been used by Pfizer to create potent positive allosteric modulators (PAM) that are extremely challenging. Novartis has also applied one of our C–H activation reactions in process chemistry to shorten steps by half. Our enantioselective C–H methylation reaction of pyrrolidine has also led to a drug candidate at Abide Therapeutics founded by my colleague Benjamin Cravatt, which has recently been acquired by Lundbeck for $400 million.

In brief, solutions to these challenges will critically depend on advances in the following three tasks: first, ligand discovery; second, ligand discovery; third, ligand discovery still.

—Jin-Quan Yu

Our C–H arylation of free alkyl amines, and C–H coupling of phenyl acetic acids have been used by Vividion Therapeutics (myself as a co-founder) to synthesize a privileged small molecule library for drug discovery. This company with many promising drug candidates has been recently acquired by Bayer for $2 billion.

From the chemical synthesis viewpoint, the most exciting application will be ton-scale production of medicines using our reactions. Optimization for scaling up our reactions in collaborations with leading pharma companies including Bristol-Myers Squibb (BMS) and AbbVie are ongoing. I am confident that one of our C–H hydroxylation reactions at room temperature will be used in ton-scale manufacture of medicines in the near future.

***NSR:*** Catalysts for C–H activation are often based on metals such as palladium or copper. Which are the most powerful metals, and why?

***Yu:*** Many other metals such as Ir, Rh and Ru have been used to study C–H activation from the 1960s to 2000s. We chose to focus on Pd(II) because our purpose is not to develop just one reaction or make one bond. Each step of synthesis may require the formation of different carbon-carbon or carbon-heteroatom bonds. Our vision from the very beginning was to develop a wide range of transformations that can be used in synthesis to avoid additional steps of interconversion. In this context, Pd(II) has the greatest potential because of its known diverse reactivity of the Pd-Carbon bonds in cross coupling.

The emergence of Cu(II) for a wide range of C–H activation reactions was one of the most unexpected surprises in the field of C–H activation. We were using Cu catalysts to investigate biomimetic C–H hydroxylation with molecular oxygen and accidentally discovered the first C–O bond-forming reaction in 2006 (this paper has been cited over 1000 times). Subsequent mechanistic exploratory studies showed that this is not a biomimetic oxidation, instead, we might have stumbled across an unexpected organometallic C–H activation reaction that is similar to our Pd(II)-catalyzed reactions. This finding has led to a systematic investigation on Cu(II) catalysts, and numerous carbon-carbon and carbon-heteroatom bond-forming C–H activation reactions have been developed from our laboratory over the past 15 years. Other laboratories have also made a number of interesting contributions with Cu(II) catalysts. However, compared to Pd(II) catalysts, we have a long way to go before we can declare victory on Cu(II) catalysts, largely because we have not found an effective ligand yet.

***NSR:*** What are the remaining challenges for C–H activation?

***Yu:*** From the practical perspective, the challenges are: (i) broadening the scope of all major classes of native substrates, from aliphatic acids, ketones, to amines and alcohols; (ii) extending the activation site of an aliphatic chain one or two carbons further away from the functional groups; (iii) increasing the catalytic turnover number to the 1000–10 000 level for a wider range of reactions; (iv) using practical oxidants such as molecular oxygen, aqueous hydrogen peroxide for a wider range of transformations.

From the intellectual perspective, we need to invent more approaches and new catalysts to increase site-selectivity and enantioselectivity.

In brief, solutions to these challenges will critically depend on advances in the following three tasks: first, ligand discovery; second, ligand discovery; third, ligand discovery still.

***NSR:*** How are Chinese scientists performing in the field of C–H activation?

***Yu:*** They are one of the major forces in the field. Some of them are unique. For example, Xiao-Guang Lei's total synthesis of (−) Incarviatone A, using multiple C–H activation reactions developed in our lab; Ang Li's asymmetric synthesis of Delavatine A, using one of our enantioselective C–H activation reactions; Bing-Feng Shi's total synthesis of (+)-Steganone, using our chiral transient directing group strategy. Gong Chen's lab has demonstrated elegant synthesis of peptide-based natural products using C–H activation reactions. Tiansheng Mei is one of the pioneers using electrochemistry to close the redox catalytic cycle of Pd-catalyzed C–H activation reactions. Meng-Chun Ye's lab has recently reported a Ni-catalyzed remote site-selective C–H activation reaction. Hui-Xiong Dai's lab has made substantial contributions to broadening the Cu(II)-catalyzed C–H activation transformations.

## PERSPECTIVES ON ORGANIC CHEMISTRY

***NSR:*** Is there any new method appearing in the field of synthetic chemistry?

***Yu:*** Organic chemists are continuing to invent new methods to address various challenges, for example, formation of complex fused rings, carbohydrate synthesis, nucleotide synthesis, catalysis using electrochemistry, and photo chemistry are still making great strides. I also anticipate copper-catalyzed C–H activation reactions we initiated in 2006 will take off in the next 10 years.

***NSR:*** Will there be universal catalysts or universal catalyst designing methods for the great diversity of substrates and reactions?

***Yu:*** ‘Universal’ is probably the wrong word for catalyst design. Even in the field of conventional double bond chemistry, it is naïve to expect a universal catalyst. Enzyme is the masterpiece of catalyst design; nature chooses to focus on extraordinary selectivity and inevitably limited substrate scope. In contrast, synthetic chemists have developed a number of catalysts that can accommodate broader substrate scope. In this case we describe this type of catalysts as broadly useful catalysts, but not universal.

As a fundamental principle, selectivity is achieved by precise molecular recognition, therefore, achieving high selectivity with different classes of substrates is unlikely to be feasible using the same catalyst. In addition, different carbon-carbon or carbon-heteroatom bond-forming reactions often require different catalyst structures. The ideal scenario will be that we strategically separate substrates into a number of major classes, and we design different classes of catalysts for them, and this is exactly how we approach C–H activation reactions. In my opinion, the gold standard for a general catalyst is the compatibility with 10 000 substrates.

Having said this, there will be some principles that can be generally applied to the design of various different catalysts. For example, our concept of using weak coordination from substrates to achieve entropic need and bifunctional ligand to boost enthalpic contribution has been broadly successful in catalyst design for different classes of substrates, for example, carboxylic acids and amines.

***NSR:*** You have described your work as ‘molecular editing’. What does that mean?

***Yu:*** Meaning to modify a molecule at any site in any order by replacing C–H bonds with other synthetically useful bonds. In this context, selective activation of a particular C–H bond is not sufficient. The ultimate challenge is to be able to stereoselectively activate multiple C–H bonds in any order to meet the need of a synthetic task at hand. In my view, molecular editing is the ultimate tool to access extreme diversity of chemical space and achieve ideal synthesis. In short, this is the Apollo 17 moonshot in organic synthesis.

***NSR:*** What are the potential approaches to ‘activate multiple C–H bonds in any order’?

***Yu:*** Considering the vast number of different substrate structures, it is wise to use various approaches. The classic approach of controlling site selectivity via electronic and steric effects is still valuable, but highly limited.

The majority of the strategically important C–H bonds are often remote from functional groups and electronically similar; therefore, it is difficult to rely on this conventional approach. If you study enzymatic catalysis in depth, you will find enzymes form multiple weak interactions with substrates to achieve remarkably site-selective catalysis. Such selectivity is often attributed to the complex binding pocket of enzymes, which has

The ultimate challenge is to be able to stereoselectively activate multiple C–H bonds in any order to meet the need of a synthetic task at hand.  . . .In short, this is the Apollo 17 moonshot in organic synthesis.

—Jin-Quan Yu

guided (or perhaps mis-guided) synthetic chemists to imitate such a magic binding pocket without considering directing effect, albeit with limited success. However, if you analyze the enzymatic site-selective remote oxidation of aliphatic acids deeply and distill out the fundamental principle, you will realize that enzyme is the master of remote directing, and the site selectivity is controlled by distance and topology of the macrocyclic transition states.

With this understanding, we have established a new approach, named ‘carpenter's approach’, in which catalytic directing templates are designed to reversibly interact with substrates and form macrocyclic transition states. The distance and geometry will dictate the energy barrier of the transition states thereby controlling site selectivity.

***NSR:*** The 2021 Nobel Prize in Chemistry was awarded to Benjamin List and David MacMillan for their development of organocatalysis. Is this work related to your research field?

***Yu:*** Organocatalysis is a significant alternative approach to asymmetric catalysis for making chiral molecules, hence, the focus is on rendering known reactions enantioselective. Typically, the reactions involve reactive functional groups, and the stereochemistry is largely established from flat pi-bonds. C–H activation focuses on inventing reactions that will offer new logic of synthetic disconnections. Rendering these C–H activation reactions enantioselective involves an entirely different stereomodel, namely, breaking the symmetry of tetrahedral carbon centers. No doubt, enantioselective C–H activation reactions will have transformative impact on asymmetric catalysis and that is one of the major challenges my laboratory has been trying to address.

## REFLECTIONS AND ADVICE: BE PASSIONATE AND ANALYTICAL

***NSR:*** It has been five years since you became one of the 2016 MacArthur ‘Genius’ Fellows. How did you use that money?

***Yu:*** It has helped me tremendously in many ways. It was not clear at all how far we could push the field forward in 2016 even though we had a few promising findings. Working on a challenging problem is highly risky including losing funding or even one's job. This grant encouraged me to really take on highly risky projects, and not be concerned about quick success in order to gain all the recognition that young scientists understandably often worry about.

***NSR:*** How do you choose your research projects? The fundamental ones, the difficult ones, the interesting ones, or the useful ones?

***Yu:*** Ideally, you want to work on a project that has all of these elements. Typically, some of these elements can overlap if you choose well. For example, our first project on chiral oxazoline directing auxiliary for asymmetric C–H activation is fundamentally important as no one has built a model system to investigate the chiral transition state for C–H cleavage, but clearly it is not

You really need tons of enthusiasm to go from failure to failure in order to carry on all the way to the final success.

—Jin-Quan Yu

useful if we stopped at that level. Since it is so interesting and will provide a key missing piece of knowledge to the field, I chose to work on it in 2002.

Our project on remote C–H activation was extremely difficult in 2009. The initial system we built was also contrived and not useful. We chose to work on it because it is not only interesting but also we could see the potential.

Our recent advance on using molecular oxygen and aqueous hydrogen peroxides are both fundamentally important and useful. These projects not only fill important scientific gaps, but also bring C–H activation reactions closer to large-scale manufacturing.

In chemistry, an important project often takes many small steps to finally advance to the level where everyone can see the importance and utility. Many of our projects and approaches have been widely criticized as being contrived until this day. For example, the typical criticism is the use of a directing group because everyone is still mis-guided by the confusion of non-directed activation of methane or alkane with reactions of synthetic substrates. It is crucial to analyze big challenges and solve the problems step by step as long as you have the long-term vision.

***NSR:*** How does the COVID-19 pandemic influence your research?

***Yu:*** I had less travel, fewer meetings and more time for thinking, which turned out to be not a bad thing. Several good ideas came to me this year [2021] and they have led to very exciting discoveries. One of the major concepts for catalyst design, namely, to gain dual ligand function via tautomerization, has led to the first C–H hydroxylation with molecular oxygen. This work has recently been published in *Science*.

Certainly during this pandemic it has also struck me that I should put more emphasis on how to use our new reactions for drug discovery. Scripps has an excellent small molecule drug discovery program targeting COVID-19, and I am remotely involved to provide any assistance they may need.

***NSR:*** You kept a distinguished curly big hairstyle for years. When and why did you start that?

***Yu:*** Mainly because it cost money and time to get a haircut when I was a student at Cambridge. When I was finally promoted to a full professor at Scripps, I started to get a haircut service more often and I realized that none of my colleagues were happy with my short hairstyle. In fact, my department chair Professor Nicolaou called me to his office and had a serious conversation about this, I remember he said: ‘Jin-Quan, can you do me a favor by keeping your hairstyle, because that is a signature style that goes well with your crazy chemistry’. My close friend Phil S. Baran, another remarkable synthetic chemist, once joked: ‘You must keep your hairstyle, otherwise, your H-index (hair-index) will not grow as fast as it did in the past 10 years’.

***NSR:*** You are very energetic and passionate, and many other successful scientists have a similar disposition. Do you think it is necessary for a scientist to be passionate?

***Yu:*** Yes, for any significant scientific problem in chemistry, it is not a 100-meter dash, but a marathon. It is unlikely that anyone can solve a big problem by working in a certain field for three or five years. You really need tons of enthusiasm to go from failure to failure in order to carry on all the way to the final success.

***NSR:*** During your scientific career, have there been any important events or people that have significantly influenced you?

***Yu:*** Many. My master's degree advisor, Professor Shude Xiao, afforded me great confidence by putting enormous trust in me to design experiments. My Ph.D. advisor, the late Dr JB Spencer, was a very special friend like a brother; he greatly shortened the time it took for me to understand the Western world of science and life at that time. My postdoctoral advisor, Professor EJ Corey, is an extraordinary giant in organic chemistry and a remarkable person who has advised me at every step of my career like a father. It is fair to say, his influence was crucial when I proposed my first independent project in 2002. I still remember clearly, when I showed him a rough draft of my proposal, he made a red exclamation mark on the top, and just said one sentence: stick to this idea!

***NSR:*** What are your suggestions for young chemistry researchers?

***Yu:*** Focus, be passionate, be self-critical, and above all, be brutally honest and analytical.

We all know phrases like ‘thinking outside the box’ or ‘from 0 to 1’, but you need outstanding analytical ability to figure out, what is the box? What is inside the box and what might be outside the box? What is a meaningful ‘0 to 1’?

